# An optically driven microstructure for torque measurement in rotary molecular motors

**DOI:** 10.1038/s41378-026-01185-5

**Published:** 2026-02-03

**Authors:** Giacomo Donini, Silvio Bianchi, Nicola Pellicciotta, Giacomo Frangipane, Maria Cristina Cannarsa, Ojus Satish Bagal, Roberto Di Leonardo

**Affiliations:** 1 Department of Physics, ‘Sapienza’ University of Rome, Rome, Italy; 2 NANOTEC-CNR, Institute of Nanotechnology, Soft and Living Matter Laboratory, Rome, Italy

**Keywords:** Micro-optics, Optical materials and structures

## Abstract

‘Light-mills’ are optically driven microstructures that can exchange orbital angular momentum with light and thus rotate around a central axis with a controlled applied torque. Although many studies have explored the employment of light momentum for torque generation, only a few convincing applications in cellular and molecular biology have been demonstrated. Here, we design a 3D chiral structure that can be selectively coupled to a target nanometric flagellar motor in a live E. coli cell, functioning as an external, tunable torque clamp. We optimize our 3D microstructures for torque conversion efficiency and mechanical stability, and propose a calibration protocol that enables absolute quantification of the torque generated by the flagellar motor during rotation in both its natural and reverse directions. Our results demonstrate that microfabricated light-mills expand the optical toolbox for biomechanical study of individual rotary motors by enabling controlled torque application and measurement at the nanoscale.

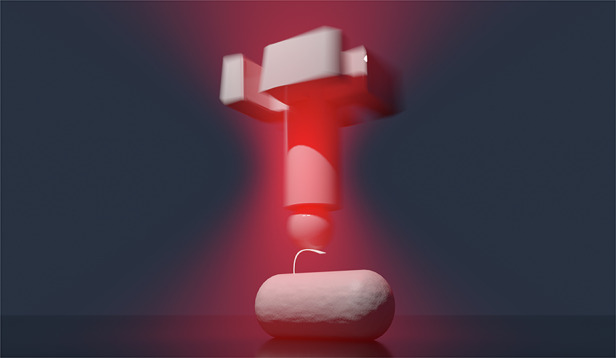

## Introduction

From duplication to transport, energy harvesting, and cell propulsion, cellular function relies on a large number of enzymes that convert chemical or electrochemical energy into mechanical work and are therefore referred to as molecular motors. While most experiments characterize force and displacement, torque generation and rotational motion play an equally important role in many of these machines. For this reason, significant efforts have been made in recent years to develop single-molecule techniques for torque spectroscopy^1,2^. These studies span systems from RNA polymerase^3,4^ to kinesin^5^, and especially to rotary motors. The small (but perhaps growing^6^) subclass of rotary motors includes the F_1_F_*o*_-ATPase^7^ and the bacterial flagellar motor (BFM)^8^, two fundamental multisubunit membrane proteins that use the proton electrochemical gradient^9–13^ to drive rotation—which, in the first case, results in ATP synthesis, and in the latter, in cell locomotion and chemotaxis^8,14^. Given its biological relevance, the BFM has been extensively studied in past and recent literature^14,15^. The bead assay is the most widely used technique for studying flagellar motors in vivo^15^. In this assay, the rotation of a single motor is coupled to that of a polystyrene microsphere, whose coordinates can be easily measured. This method has provided key insights into various aspects of motor function, such as the reversal of rotation direction associated with chemotaxis^16–18^ and the mechanosensitive dynamics of torque-generating proteins^19–22^. Additionally, the bead assay enables torque measurements by calculating the drag exerted on the bead and multiplying it by the motor’s rotational speed^12,23,24^. By altering the drag, either through changing the viscosity of the medium^25,26^ or using beads of different sizes^23,27^, torque can be measured for different rotational frequencies.

An alternative approach involves applying an external torque to the motor. Coupling superparamagnetic beads to the motor, for instance, allows for easy stalling using permanent magnets^20,24,28^, or even control of its rotational frequency via a rotating magnetic field^29^. Although promising, the latter approach remains underexplored, and a calibration procedure is still lacking for its use in torque measurements. In a similar manner, rotating electric fields produced by four electrodes can rotate a tethered cell^30–34^. While this technique can generate large torques, it faces significant calibration challenges, and torque values are not provided in absolute terms.

Finally, optical manipulation at the micro- and nanoscale is a very promising approach offering high precision, high spatio-temporal resolution and non-invasiveness. Many techniques are being developed to apply optical torques both for optical sorting^35^ and torque spectroscopy^2^. Optical tweezers are a valuable option for micrometer-scale manipulation and force measurement^36^. Despite their potential for studying flagellar motors, they have so far provided mainly preliminary data^37^. One of the main challenges arises from the fact that, while optical tweezers can apply a force, the resulting torque is difficult to determine without knowing the moment arm^38^. An alternative strategy within the realm of optical manipulation involves exploiting the angular momentum carried by light^39^. Whenever an object changes the angular momentum of a beam, it will be subject to a torque given by the exchanged angular momentum per unit time. In birefringent particles such as vaterite microspheres^40,41^, the spin component of optical angular momentum is exploited^41–44^. For example, a particle changing the polarization of a beam from left circular to right circular undergoes a recoil torque of *Pλ*/(*πc*), where *P* is the power and *λ* the wavelength^43^. Alternatively, it is possible to spin a birefringent particle while simultaneously controlling its center position using an optical tweezer, by rotating the linear polarization of the trapping laser. By detecting the change in angular momentum of the transmitted trap beam it is also possible to measure the torque applied to the particle^45–47^. This system is usually referred to as an optical torque tweezer or optical torque wrench and it has been proved to be useful for the study of biophysical systems at the molecular level^5,48,49^. Nonetheless, to date, no application of this technique to the BFM has been described in the literature. Other approaches to exploit spin angular momentum include the use of absorbing particles^50^, which, however, lead to undesirable heating effects in biological investigations. Alternatively, it is theoretically possible to exert an optical torque on chiral spheres^51,52^, although the expected magnitude is extremely small and the preparation of such particles is highly challenging^53,54^. Finally, it has been recently demonstrated that it is possible to generate and measure an optical torque on non-absorbing spheres by using a spin-gradient light field^55^. This technique presents several advantages, yet the torque produced is about three orders of magnitude smaller than that generated by the BFM.

As an alternative to spin angular momentum, it is possible to exploit orbital angular momentum of light to rotate microscopic chiral objects called light-mills^56–62^. Objects of chiral shapes can indeed impart a net angular momentum to scattered light, generating a recoil torque that can be precisely controlled by modulating the power of the illuminating beam^63^. Although this approach shows great potential, so far it has only been demonstrated in controlled experimental settings, with no applications in a real biological context.

In this paper, we introduce a novel assay for studying the bacterial flagellar motor. We design a light-mill^59–62^ structure that can be positioned using optical tweezers, while the optical torque can be adjusted by controlling the trapping beam’s power. Prior to attachment to the flagellar motor, the light-mill can be characterized in terms of drag and torque conversion. Using this method, we probe the torque produced by the motor when rotating in both its natural direction (counterclockwise, CCW), stalled, or driven backwards (clockwise, CW) by the external optical torque. We show that the motor generates the same torque regardless of the direction of rotation, and we demonstrate that the motor’s rotational direction can be inverted smoothly and reversibly.

## Results and discussion

### Free structure

**Fig. 1:**
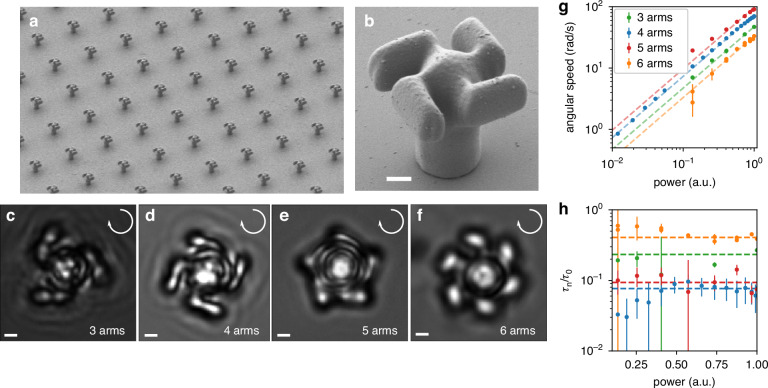
Light-mill design and performance. **a** An array of light-mill structures. **b** Close-up view on a single light-mill. **c**–**f** Brightfield images of light-mills. The four-arm design (**d**) is shown along with variants with three (**c**), five (**e**) and six (**f**) arms. The structures are held in position by an optical trap but are free to rotate around their axis. All scalebars are 1 *μ*m. **g** Angular speed of the light-mills in (**c**–**f**) as a function of the laser power. **h** Ratio between the oscillating coefficient and the constant term of the optical torque (see Eq. (2)) as a function of power

Figure 1a shows a SEM image of an array of our microfabricated light-mills, with a zoomed-in view of one of the structures shown in Fig. 1b. Structures are made of SU8 photoresist and are fabricated using a two-photon polymerization technique^64,65^. The central cylinder (5 *μ*m tall, 1 *μ*m radius) of the structure guarantees a stable three-dimensional trapping, while minimizing fluctuations in its tilt angle. The top of the structure consists of four arms, giving the structure a chiral shape (the radius from the center to the arm tip is 2.4 *μ*m).

We first test how our light-mills rotate in the bulk fluid. We wet the structures with deionized water and detach them using a tapered glass tip controlled by a micromanipulator. Once a structure is separated from the coverslip, we trap it with a single focused IR beam (*λ* = 1064 nm). Our trap is controlled by a Spatial Light Modulator (SLM), which is placed in the Fourier plane with respect to the objective lens^66,67^. A linear phase modulation is displayed on the SLM to produce a single spot that, in the sample plane, is displaced by 15 *μ*m from the 0th order, with the latter blocked using an iris diaphragm. The SLM is also used to modulate the power of the trapping beam while keeping the laser power constant. We do this by intentionally reducing the dynamic range of the SLM phase modulation^68,69^. The full dynamic range of the phase mask producing the displaced trap is [0, 2*π*]. We multiply the phase mask by a factor *A*, where *A* is a number between 0 and 1, so that the modulation dynamic range becomes [0, 2*πA*], thereby reducing the diffraction efficiency in a controlled manner. The relative power of the first-order spot is *P* = sinc(*π*−*πA*)^2^ ^68,69^.

**Fig. 2:**
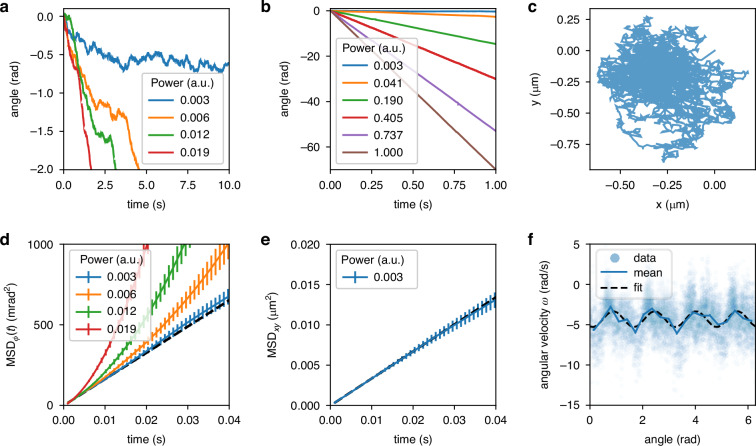
Calibration. Experiments with a four-arm light-mill trapped in bulk fluid. **a**, **b** Angle as a function of time for different values of the laser power. Brownian fluctuations are clearly visible when laser power is low. **c** *x*−*y* trajectory of the light-mill structure for *P* = 0.003 **d** Mean squared displacement of the angle relative to the tracks in (**a**). The black dashed line represents a linear fit of the blue curve for *t* < 0.01 s with angular coefficient 2*k*_*B*_*T*/*γ*_*ϕ*_. **e** Translational mean squared displacement on the *x*−*y* plane for short times. The black dashed line plots a linear fit with angular coefficient 4*k*_*B*_*T*/*γ*_*xy*_. **f** Derivative of the angle for *P* = 0.05. The blue line is the average of *ω* within small *ϕ* intervals. Black dashed line is a fit to Eq. (2)

When the light-mill is trapped, it starts to rotate. We can extract its rotation angle *ϕ* using a custom tracking algorithm based on template matching^70,71^. Figure 2a, b shows *ϕ* as a function of time for different values of power *P*. When the laser power is low, the angular velocity is low and Brownian fluctuations of the angle are clearly visible (Fig. 2a). These fluctuations can be used to extract the rotational drag *γ*_*ϕ*_, of the light-mill. For each power *P*, we compute the mean square displacement $MS{D}_{\phi }(t)=\langle {[\phi ({t}^{{\prime} }+t)-\phi ({t}^{{\prime} })]}^{2}\rangle$. The MSD is the superposition of a diffusive component and a quadratic component due to linear drift:

1 $$MS{D}_{\phi }(t)=2Dt+{\langle \omega \rangle }^{2}{t}^{2}$$

where *D* = *k*_*B*_*T*/*γ*_*ϕ*_ is the diffusion coefficient and 〈*ω*〉 the time-averaged velocity. A few MSDs are plotted in Fig. 2d. When the power is sufficiently low, the diffusive term is dominant on short times, so that the MSD can be fitted to extract *γ*_*ϕ*_ = 0.50 ± 0.02 pN *μ*m s. The translational drag can be extracted in a similar way. For low laser power, the trapped light-mill position fluctuates around a stable point as shown in Fig. 2c where the plotted *x* − *y* trajectory shows the fractal pattern typical of Brownian motion. We then compute $MS{D}_{xy}(t)=\langle {[x({t}^{{\prime} }+t)-x({t}^{{\prime} })]}^{2}+{[y({t}^{{\prime} }+t)-y({t}^{{\prime} })]}^{2}\rangle$. As shown in Fig. 2e, for short times the MSD is approximately *MSD*_*xy*_(*t*) ≈ 4*Dt*, where *D* = *k*_*B*_*T*/*γ*_*xy*_ is now related to the translational drag coefficient whose fitted value is *γ*_*xy*_ = 0.049 ± 0.002 pN *μ*m^−1^ s.

The optical torque is proportional to the laser power, which means that the rotational velocity is also proportional to *P*, as shown in Fig. 1g. However, the instantaneous velocity $\omega =\mathop{\phi }\limits^{^\circ }$, is not uniform during the rotation, as depicted in Fig. 2f, which plots *ω* as a function of *ϕ*. Brownian motion and tracking error cause substantial noise in the single data point. A smooth curve can be obtained by averaging the data points over small *ϕ* intervals, plotted in Fig. 2f as a blue solid line. The oscillations of *ω* around a constant value are clearly visible. Both terms, the constant one and the oscillating one, grow linearly with *P* so that

2 $$\gamma_\phi \omega (\phi )={T}_{{\rm{opt}}}(\phi )=-P\left({\tau }_{0}+{\tau }_{4}\cos (4\phi )\right)$$

where *τ*_0_ and *τ*_4_ are power-to-torque conversion factors. Fig. 1h plots *τ*_4_/*τ*_0_ as blue points. The average value for the *τ*_4_/*τ*_0_ ≈ 0.07 is shown as a horizontal dashed line. With this value of *τ*_4_/*τ*_0_ it can be verified by integrating Eq. (2) that the time-averaged velocity 〈*ω*〉 deviates from the angle-averaged velocity $\overline{\omega }=-P{\tau }_{0}/\gamma$ by less than 0.5%. The oscillations in *T*_opt_ are due to the four-fold symmetry of the structure. When the trapping beam is circularly polarized, it is invariant under rotations, so one would expect that a light-mill centered on the beam experiences the same torque *T*_opt_ independently of the angle *ϕ*, due to the rotational symmetry of the system. We use a quarter-wave plate to circularly polarize the trapping beam, but we observe no significant improvement in the ratio *τ*_4_/*τ*_0_ with respect to the corresponding uncertainty (about 10%). If spatial aberrations are present in the trapping beam, the rotational invariance is broken, leading to an angle-dependent torque. Although these aberrations were minimized using the SLM, small residual distortions may still remain. As we will show later, completely eliminating these small oscillations is unnecessary, since connecting the light-mill to the bacterial motor will inevitably result in misalignment between the motor and light-mill axis.

We also tested different light-mill designs by varying the number of arms. Figures 1c–f show brightfield snapshots of light-mills with three (c), four (d), five (e) and six arms (f). The average angular speed as a function of *P* for the light-mills is shown in Fig. 1g. At a given power, the rotational velocities of the light-mills are in the same order of magnitude, with the five-armed light-mill spinning slightly faster. For the four-armed light-mill, the oscillating term in Eq. (2) is ${\tau }_{4}\cos (4\phi )$; similarly, for an n-armed light-mill, the corresponding term is ${\tau }_{n}\cos (n\phi )$. Figure 1h plots the ratios *τ*_*n*_/*τ*_0_ for *n* = 3, 4, 5, 6. The four-armed light-mill exhibits smoother rotation, *that is*, a smaller *τ*_*n*_/*τ*_0_, and was therefore selected for the subsequent experiments.

### Torque-speed measurement of the BFM

**Fig. 3:**
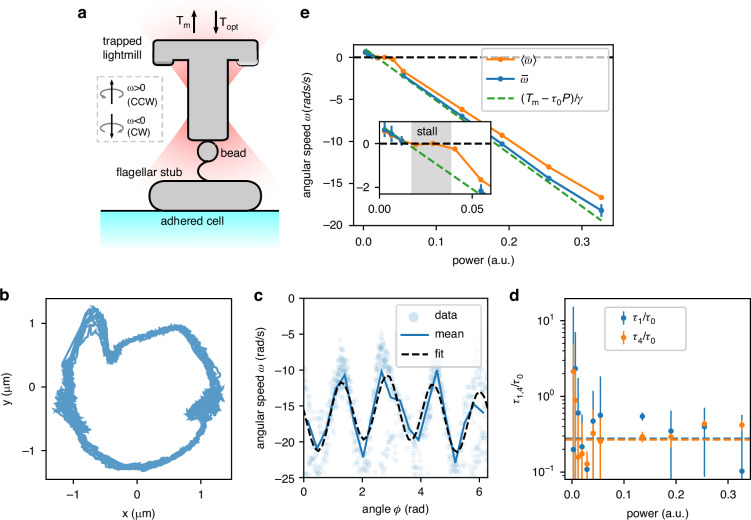
Coupling to the bacterial flagellar motor. **a** Sketch of the experimental setup. A four-arm light-mill is attached to the motor through a 1.6 *μ*m bead. **b** Trajectory in the *x* − *y* plane of an attached light-mill rotating around the flagellar motor axis at a radius *r* ≈ 1*μ*m. **c** Instantaneous angular velocity *ω* as a function of the angle and for *P* = 0.32. The black dashed line represents a best-fitting curve as explained in the text. **d** Values of *τ*_1_/*τ*_0_ and *τ*_4_/*τ*_0_ (see Eq. (5)) as a function of power. **e** Orange dots plot the time-averaged rotational speed 〈*ω*〉 as a function of the laser power. Blue dots plot the angle-averaged speed $\overline{\omega }$. The dashed green line is the expected curve if we assume that the motor torque is constant and equal to *T*_*m*_ = *T*_free_ (estimated from the motor rotating a bead). The light-mill drag *γ* and the power to torque coefficient *τ*_0_ are those obtained from calibration performed before the light-mill was attached

Although the surface of the light-mill is hydrophobic, we cannot attach it directly to a flagellar stub as the latter is not visible under bright-field microscopy. Conversely, as in the standard bead assay^15^, when a bead is observed rotating around a fixed center, there is clear evidence that it is attached to a flagellar motor via a strong hydrophobic interaction. We use this criterion to identify properly attached beads and use them as targets for light-mill attachment. When the light-mill and the bead come into contact the two adhere rigidly and irreversibly because of Van der Waals interactions^72^. To check light-mill to motor coupling, we turn off the optical trap and verify that the light-mill is driven by the flagellar motor to rotate anticlockwise (see Supplementary Video 1). A sketch of the experiment is shown in Fig. 3a. We use a strain lacking the response regulator gene cheY, so that the BFM is locked in the CCW state and thus the torque *T*_*m*_ does not change sign with time. The structure chirality is such that the optical torque (*T*_opt_ < 0) has opposite sign with respect to the one provided by the motor (*T*_*m*_ > 0):

3 $$\gamma \omega ={T}_{{\rm{opt}}}+{T}_{m}$$

where *γ* is the drag of the light-mill. Before attaching the light-mill to a rotating bead, we measure the bead's angular velocity *ω*_free_, which allows us to estimate the torque of the ‘freely’ rotating flagellar motor as *T*_free_ = *ω*_free_*γ*_*b*_, where *γ*_*b*_ is the drag of the bead (see Materials and methods). For the experiment shown in Fig. 3, we have *ω*_free_ = 86 ± 6 rad s^−1^ and *γ*_*b*_ = 0.011 ± 0.001 pN *μ*m s, yielding *T*_free_ = 1.1 ± 0.2 pN *μ*m. We also calibrate the light-mill in the bulk, as shown in the previous section, obtaining *τ*_0_, *γ*_*ϕ*_ and *γ*_*xy*_. These parameters will be used in the analysis of the assembled system to derive *T*_*m*_ from Eq. (3).

After the attachment, the axis of the flagellar motor and the axis of the light-mill are never perfectly aligned. The compliance of the flagellar stub, which acts as a torque-transmitting joint^73^, allows the light-mill to be rotated by the motor while the former is held by the trap. The position of the light-mill relative to the trap is not that of the free structure. Fig. 3b shows the *x* − *y* position of the light-mill rotating around the motor axis. The mean radial distance from the axis is *r* = 1.0 ± 0.2 *μ*m (where ± 0.2 here indicates the standard deviation). We then estimate the total drag *γ* of the mounted light-mill as

4 $$\gamma ={\gamma }_{\phi }+{\gamma }_{xy}{r}^{2}+{\gamma }_{b}$$

where the dominant contribution to *γ* = 0.56 ± 0.03 pN *μ*m s is given by *γ*_*ϕ*_, while the sum of the two remaining terms contributes with a correction of about 10%. The rotation of the light-mill *x* − *y* position respect to motor also results in an increased dependency of *T*_opt_ on *ϕ*:

5 $${T}_{{\rm{opt}}}=-P\left[{\tau }_{0}+{\tau }_{1}\cos (\phi +{\delta }_{1})+{\tau }_{4}\cos (4\phi )\right]$$

where *τ*_1_ is a power-to-torque coefficient and *δ*_1_ is a phase. This means that in addition to the oscillations due to the four-fold symmetry, we observe another term arising from the structure rotating around an axis that does not coincide with that of the trapping beam. Fig. 3c shows *ω* as a function of *ϕ* for *P* = 0.33. Compared to the free case Fig. 2f, the attached light-mill now shows larger oscillations in torque. These oscillations are estimated from *ω* and plotted in Fig. 3d.

The orange points in Fig. 3e plot the time-averaged angular speed as a function of the laser power. When power is low, the motor torque is larger than the optical torque, and the structure rotates counterclockwise (〈*ω*〉 > 0). Conversely, when *P* is such that the optical torque dominates, the structure rotates clockwise (〈*ω*〉 < 0). Additionally, there is a range of laser power values for which the motor stalls.

**Fig. 4:**
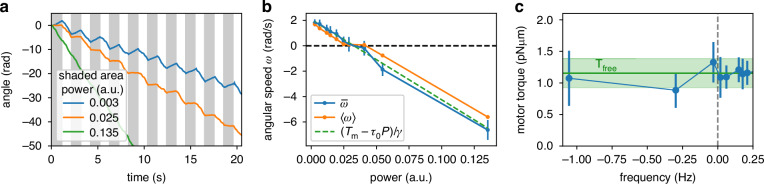
Torque-speed measurement. **a** Angle as a function of time when the laser power is alternated between *P*_r_ = 0.14 (unshaded area) and a test power *P* (shaded area). Whenever the power is switched back to *P*, *ϕ* starts from a `random' value, which is obtained by when the light-mill is rotated rapidly backward. **b** Time and angle averaged velocities as a function of *P*. Averages are computed only over the data points belonging to the shaded areas in (**a**). The dashed green line plots the expected curve for a constant motor torque as in Fig. 3e. **c** Torque-speed curve obtained starting from the angle averaged velocity $\overline{\omega }$ in (**b**). Horizontal line is the torque *T*_free_ measured before the light-mill is attached, while the shaded area represents its confidence interval

If we used Eq. (3) to find *T*_*m*_ as a function of *ω*, we would observe a discontinuity in the torque at *ω* = 0. A similar result was observed in electrorotation experiments^31,32^. As argued by the same authors, this result is an artifact caused by a non-constant value of the external torque^33^. This is indeed the case for our light-mill as well. The fact that 〈*ω*〉 = 0 over a range of laser power values (Fig. 3e, insert) can be explained by combining Eq. (3) and Eq. (5): the angle *ϕ* evolves to a stable point where *ω* = 0 if *P* lies approximately in the range *T*_*m*_/(*τ*_0_ + *τ*_1_ + *τ*_4_) < *P* < *T*_*m*_/(*τ*_0_ − *τ*_1_ − *τ*_4_), *that is*, when the constant term ∣*T*_*m*_ − *Pτ*_0_∣ is smaller than the amplitude of the oscillations, which is approximately *Pτ*_1_ + *Pτ*_4_. To avoid potential artifacts and have a much simpler analysis, we rather look at the angle-averaged speed. Eq. (3) simplifies to

6 $$\gamma \overline{\omega }={T}_{m}-P{\tau }_{0}$$

where $\overline{\omega }$ the rotational speed averaged over the angle *ϕ*. Blue points in Fig. 3e plot $\overline{\omega }$ as a function of *P*. When the structure does not rotate, *ϕ* is sampled in a small range and $\overline{\omega }$ cannot be evaluated. To work around the problem, we alternate between two laser power values: the first one is varied for each acquisition, the second one is a fixed large value that is used to quickly rotate the structure so that a new random value of *ϕ* is reached. Figure 4a shows *ϕ* as a function of time for three different values *P*. The angle is ‘randomized’ at the end of each unshaded area where the power is always *P*_r_ = 0.14. When the power is switched back to the target P value (shaded areas), the motor quickly recovers its previous state. Both 〈*ω*〉 and $\overline{\omega }$ are computed for the data points relative to the shaded areas in Fig. 4a and then plotted in Fig. 4b. Again, the time-averaged velocity shows an apparent discontinuity, while $\overline{\omega }$ displays a regular behavior. Our data compares well with the green dashed line, which represents the expected angle-averaged speed with a constant motor torque: $\overline{\omega }=\left({T}_{m}-{\tau }_{0}P\right)/\gamma$, where the parameters *γ*, *τ*_0_ and *T*_*m*_ are those measured before the light-mill is attached to the motor. We invert Eq. (6) and use the calibrated values of *γ* and *τ*_0_ to extract the motor torque as a function of *ω* and plot it in Fig. 4c. Data show that *T*_*m*_ is constant even when the motor is forced to rotate forward or backward, a measurement that is in agreement with what has been found previously^38^. Our measurement is reported in absolute terms and thus can be compared in Fig. 4c with the ‘free’ motor torque, that is, the torque provided by the motor to a passive viscous load (a polystyrene bead).

## Conclusion

In this study, we demonstrate that a light-driven micro-rotor can be coupled to the bacterial flagellar motor, providing a tunable torque clamp. By adjusting the laser power, we can apply a controlled resisting torque to the flagellar motor, forcing it to rotate backwards at higher powers. We show that the motor torque remains constant for both small positive and negative rotational frequencies. Through an accurate calibration of the power-to-torque conversion before attachment, we achieve direct motor torque measurements in absolute values. To overcome artifacts arising from unavoidable misalignments between the light-mill, trapping beam, and flagellar motor, we implement a phase-averaging approach, in which data is acquired during cycles of alternating laser power. Our method demonstrates that the orbital angular momentum of light can provide new tools for quantitatively investigating the biomechanics of molecular motors. The fixed torque capabilities of the presented tool, along with the inherent ability to trap and independently drive multiple rotors, expand the current possibilities of existing torque spectroscopy techniques based on optical manipulation and magnetic tweezers.

## Materials and methods

### Microfabrication

Light-mills are fabricated on soda lime glass substrates pre-cleaned for 24 hours in a solution of sulfuric acid (95–98%) and NoChromix reagent (5% w/v), then rinsed thoroughly in deionized water. SU-8 photoresist (300 *μ*l) is spin-coated first at 500 rpm for 10 s, then at 2000 rpm for 30 s, and soft-baked at 95 °C for 30 min, resulting in a 25 *μ*m thick layer. The microstructures are fabricated using a custom-built two-photon polymerization (TPP) setup^70,74^ with a laser scanning speed of 40 *μ*m/s at 6 mW power, arranged in arrays spaced by 30 *μ*m. After exposure, samples undergo post-exposure baking at 95 °C for 8 min, development in SU-8 developer (KAYAKU Advanced Materials) for 15 min, nitrogen drying, and 20 min plasma cleaning^75^.

### Cell culture

We used the E. coli strain PL4 (HCB1826)^19^, which has a deletion of the cheY gene, resulting in a lack of the tumbling mechanism in the cells. This strain expresses a ‘sticky’ mutation of the FliC protein, so that a flagellar stub adheres to a microsphere through idrophobic interaction. Bacteria from frozen glycerol stock were streaked on a Petri dish containing 1.5% agar and lysogeny broth (LB: 1% tryptone, 0.5% yeast extract and 0.5% NaCl). A single colony was inoculated into LB and grown overnight in a shaking incubator at 30 °C and 150 rpm. The overnight culture is diluted 100-fold into 5 mL of the previous medium, grown at 30 °C, 150 rpm. At OD ≈ 0.6, cells are collected by centrifugation (1300 rcf, 5’). The resulting pellet is washed twice by centrifugation (1300 rcf, 5’) with deionized water buffer, with 66 mM NaCl, 1 mM photassium phosphate (pH 7.0) and 0.1 mM EDTA. The flagellar filaments are sheared by passing the bacteria back and forth 60 times between two syringes (23-gauge) joined by a thin piece of polyethylene tubing. The bacterial concentration is finally adjusted to the working OD ≈ 3.

### Sample preparation

The sample is prepared as follows: light-mills are fabricated in the center of a coverglass. Then, by cutting out a few millimeters square from another coverglass and using 26 *μ*m thick double-sided tape, we create a small chamber next to the area where the light-mills were built. We then follow a procedure similar to that used for the bead assay^19^. First, we fill the chamber with poly-L-lysine (0.01% in deionized water). After 5 min we wash the chamber with motility buffer, using some blotting paper to create a flux. We then add bacteria and wait a few minutes for a layer of cells to adhere to the glass, then we wash again with motility buffer and add the 1.59 *μ*m polystyrene beads (diluted in deionized water, 0.1% volume fraction). After a few minutes, when it is possible to see a good number of beads rotated by motors, we flush the diffusing ones. We add large drops of motility buffer on the sides of the channel to prevent evaporation and to also wet the light-mills. Then, we select a light-mill and detach it from the glass using a tapered glass tip controlled by a micromanipulator. We trap the light-mill and calibrate it as previously described. After calibration, we bring it into the channel. Finally, among the beads rotated by flagellar motors, we choose the most planar one and attach the light-mill to it approaching the bead vertically, as shown in Supplemental Video 1.

### Uncertainty estimation

The uncertainty on *τ*_*n*_/*τ*_0_ plotted in Figs. 1 and 3 is obtained from the least-squares fitting routine. For instance, *ω*(*ϕ*) shown in Fig. 2f is fitted to the function $\omega (\phi )={\omega }_{0}+{\omega }_{4}\cos (4\phi )$, with *ω*_0_ and *ω*_4_ being the free parameters. From Eq. (2), we obtain *ω*_4_/*ω*_0_ = *τ*_4_/*τ*_0_, whose uncertainty is derived from the errors on *ω*_0_ and *ω*_4_. The value of *τ*_0_ is calculated as *τ*_0_ = *ω*_0_*γ*_*ϕ*_, and its uncertainty is obtained through error propagation.

The error bars on the MSDs shown in Fig. 2 are computed by dividing a 10 s trajectory into 20 subtrajectories of 0.5 s each. For each subtrajectory, we compute the MSD and then calculate the mean and its corresponding standard error. Each MSD curve is also fitted to extract a set of *γ*_*ϕ*_ or *γ*_*xy*_ values, from which we compute the mean and standard error.

The rotational drag *γ*_*b*_ of the polystyrene bead was estimated following the method described in ref. ^76^. The dominant source of uncertainty arises from the bead radius, which has a 10% relative error as specified by the manufacturer.

Error bars for $\bar{\omega }$ in Figs. 3 and 4 represent the standard error of the mean. The uncertainty in *T*_*m*_ was estimated by propagating the errors through the inversion of Eq. (6)

## Supplementary information

Supplementary Video 1
